# Allopurinol-Induced Eosinophilic Colitis as the Dominant and Initial Manifestation of Drug Reaction With Eosinophilia and Systemic Symptoms (DRESS) Syndrome

**DOI:** 10.7759/cureus.110770

**Published:** 2026-06-13

**Authors:** Sérgio Costa Monteiro, Raquel Vieira, Sara Bravo, Jorge Henriques

**Affiliations:** 1 Internal Medicine, Unidade Local de Saúde da Região de Aveiro, Aveiro, PRT

**Keywords:** allopurinol, diarrhea, dress syndrome, eosinophilic colitis, hypovolemic shock

## Abstract

Drug reaction with eosinophilia and systemic symptoms (DRESS) is a rare, potentially life-threatening drug-induced hypersensitivity reaction. Although allopurinol is a well-recognized trigger and visceral involvement is common, gastrointestinal manifestations, particularly severe eosinophilic colitis as an initial presentation, are exceptionally uncommon. We report the case of a 70-year-old woman admitted in hypovolemic shock due to acute, profuse, non-bloody diarrhea. An extensive infectious workup was negative. Diagnostic clarification was achieved through colonoscopy, which demonstrated diffuse colonic inflammation, and histology revealed marked eosinophilic infiltration consistent with eosinophilic colitis. These findings, together with mild peripheral eosinophilia, acute kidney injury secondary to dehydration, and a subtle cutaneous rash, raised strong suspicion for DRESS syndrome. The diagnosis was supported by the recent initiation of allopurinol. This case underscores an unusual and severe gastrointestinal presentation of DRESS and highlights the importance of considering this diagnosis in patients with refractory diarrhea and eosinophilia, even in the absence of prominent skin involvement.

## Introduction

Drug reaction with eosinophilia and systemic symptoms (DRESS) is a rare but potentially life-threatening severe cutaneous adverse drug reaction, with an estimated incidence of approximately one in 1,000 to one in 10,000 drug exposures and a reported mortality rate of 5-10%, most commonly due to severe visceral organ involvement. It is characterized by fever, cutaneous eruption, hematological abnormalities (typically eosinophilia and/or atypical lymphocytosis), and multi-organ involvement [1].

Allopurinol is one of the most frequently implicated causative agents worldwide and a well-recognized trigger of severe DRESS, particularly in patients with renal impairment or genetic predisposition, including carriage of the HLA-B*58:01 allele [2,3]. Although internal organ involvement is common, most frequently affecting the liver and kidneys, gastrointestinal manifestations are uncommon.

The pathogenesis of DRESS is complex and incompletely understood, involving drug-specific T-cell activation, viral reactivation, and eosinophil-mediated tissue injury [2,3]. Eosinophils contribute to organ damage through degranulation and release of cytotoxic proteins, cytokines, and chemokines, leading to inflammation and dysfunction across multiple organ systems [2,3]. Gastrointestinal involvement, and particularly colitis, has been only rarely reported, with limited cases described in the literature. Its true incidence remains unknown due to underreporting and the absence of systematic epidemiological studies [4].

This case contributes to the existing literature by highlighting allopurinol-induced DRESS complicated by eosinophilic colitis, an uncommon gastrointestinal manifestation. It underscores the importance of considering gastrointestinal involvement in patients with DRESS presenting with severe diarrhea and systemic compromise. Given the paucity of published cases and limited data regarding clinical course and management, this report helps address an important gap in the current understanding of the gastrointestinal spectrum of DRESS syndrome.

## Case presentation

A 70-year-old woman presented to the emergency department with a two-week history of profuse watery diarrhea, reporting up to 10-15 bowel movements per day. The diarrhea was non-bloody, non-mucoid, and non-foul-smelling. She denied fever, abdominal pain, nausea, vomiting, or recent travel. Her past medical history included hypertension and hyperuricemia, for which allopurinol had been initiated approximately four weeks prior to symptom onset.

On admission, the patient was found to be in hypovolemic shock, with a blood pressure of 80/50 mmHg, a heart rate of 110 beats per minute, and a tympanic temperature of 38.8°C. She was alert and oriented and did not require advanced airway support. She was immediately admitted to the Intensive care unit for aggressive hemodynamic stabilization, including intravenous fluid resuscitation and vasopressor support.

Physical examination was notable for signs of severe dehydration, including dry mucous membranes and poor skin turgor. A subtle, discrete, symmetric erythematous maculopapular eruption was observed on the trunk and upper extremities, with progressive confluence of lesions, which the patient reported had been present for approximately two weeks. This rash was initially considered nonspecific due to its mild extent and absence of systemic symptoms at presentation.

Initial laboratory investigations, expressed in Table 1, revealed mild peripheral eosinophilia, with an absolute eosinophil count of 1,634 cells/mcL. She had severe oligo-anuric acute kidney injury, with a blood urea nitrogen level of 213 mg/dL and a creatinine level of 6.3 mg/dL, attributed to profound volume depletion. Metabolic acidemia and hyperkalemia were also present. These abnormalities rapidly improved following fluid resuscitation and standard medical management.

**Table 1:** Summary of laboratory values

Labs	Values	Reference range
White blood cell count	13,125 cells/mcL	4,000-11,000 cells/mcL
Absolute eosinophilic count	1,634 cells/mcL	0-500 cells/mcL
Eosinophils (%)	12.4%	0-5%
Urea	213 mg/dL	19-51 mg/dL
Creatinine	6.3 mg/dL	0.5-1.1 mg/dL
Fecal calprotectin	>6,000,000 mcg/g	<50 mcg/g

An extensive infectious workup for acute and chronic diarrhea was initiated. Four stool cultures, including testing for enteric bacterial pathogens, were negative. Serial stool examinations for parasites were also negative. A multiplex polymerase chain reaction (PCR) panel for severe diarrhea, including *Clostridium difficile*, *Escherichia coli*, and *Salmonella *species, yielded negative results. Blood cultures were negative, consistent with the absence of fever or systemic infection. Fecal calprotectin was markedly elevated, suggesting significant intestinal inflammation (Table 1).

**Figure 1:**
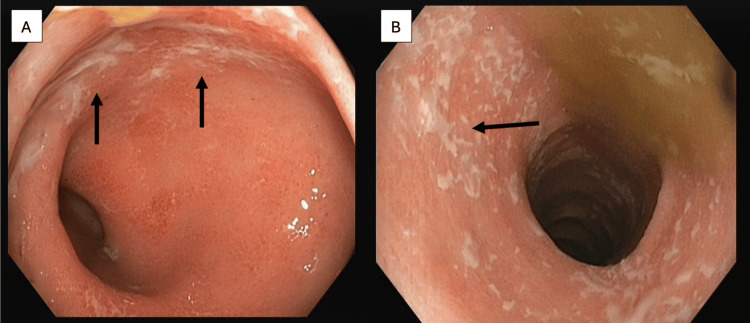
Lower endoscopy The entire colon mucosa, extending to the cecum and rectum, presents as edematous, erythematous, and eroded, with obliteration of the vascular pattern and adherent exudate. (A) Some small superficial ulcers with hematin are present in the sigmoid colon, and one is present in the proximal ascending colon (black arrows). (B) More inflammatory activity is observed in the transverse colon, ascending colon, and cecum, with superficial ulcers and erosions, some serpiginous and with granulated mucosa (black arrow).

**Figure 2:**
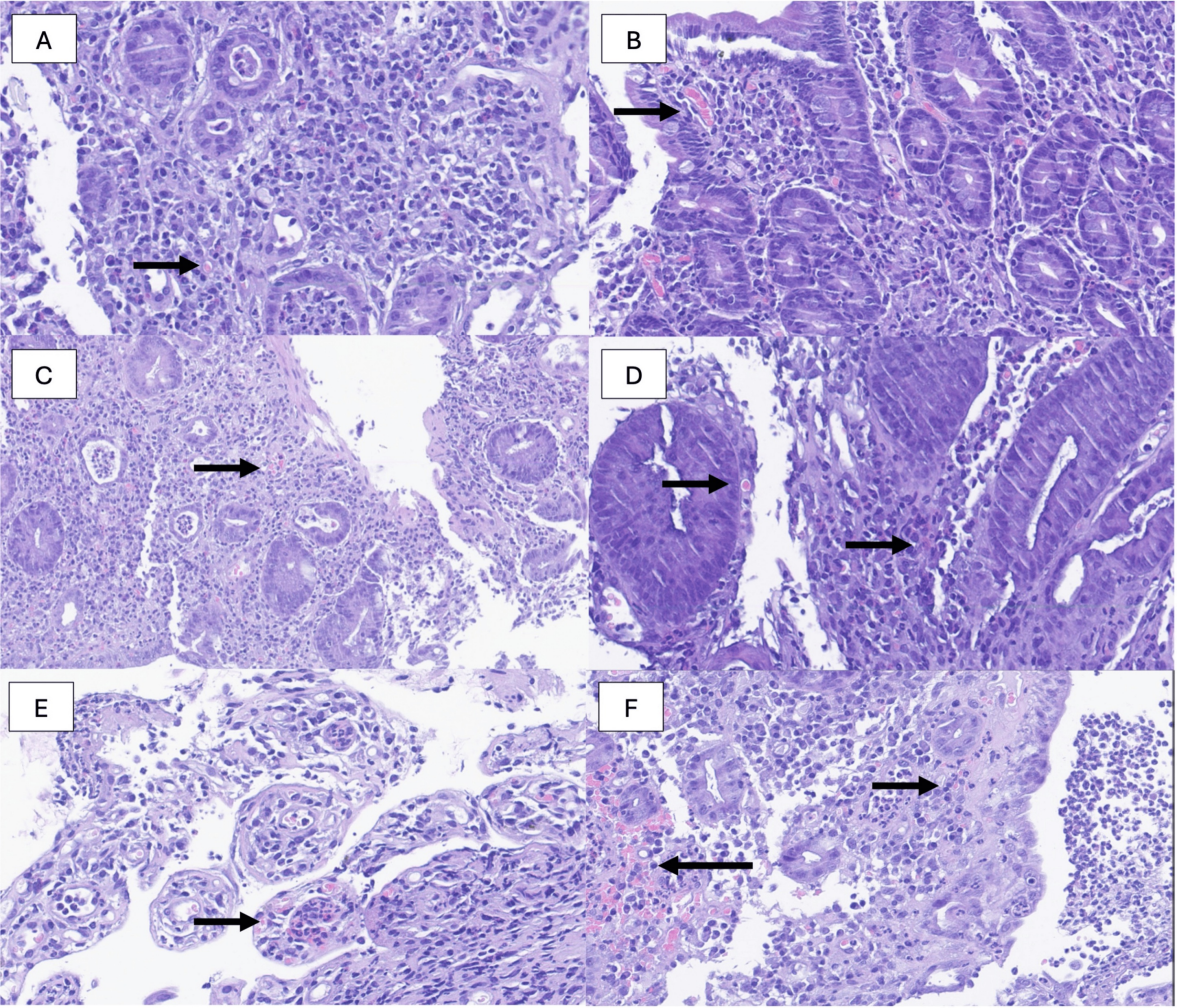
Colon and ileum biopsy: (A) cecum, (B) ileum, (C) ascending, (D) sigmoid, (E) transverse, and (F) rectum Moderate to severe ileocolitis, without specific characteristics, but which in the clinical context corresponds to enteric involvement due to a drug hypersensitivity reaction. Colonic mucosa with mild architectural distortion, foci of erosion, and reactive changes in the epithelium. The black arrows shows areas of fibrosis and abundant fibrin deposition alternate with areas of moderate to intense mixed inflammation, with frequent eosinophils, foci of cryptitis, and numerous crypt abscesses, without evidence of granulomas through all the colon and ileum. No dysplasia were documented.

After stabilization, the patient was transferred to the internal medicine ward for further evaluation of refractory diarrhea. Upper gastrointestinal endoscopy revealed no abnormalities. Lower gastrointestinal endoscopy (Figure 1) demonstrated diffuse, nonspecific inflammatory changes throughout the colon. Histopathological examination of colonic biopsies showed marked architectural distortion and a dense inflammatory infiltrate in the lamina propria rich in eosinophils, with eosinophilic cryptitis and eosinophilic crypt microabscesses, consistent with severe eosinophilic colitis (Figure 2). The pathologist reports that the density of eosinophils exceeds normal site-specific values, with 40-50 eosinophils per high-power field. PCR reaction testing for cytomegalovirus and herpes simplex virus types 1 and 2 on biopsy specimens was negative.

A thorough review of the patient's medication history revealed that allopurinol had been initiated four weeks prior to the onset of symptoms. Given the combination of eosinophilic colitis (visceral organ involvement), peripheral eosinophilia, subtle cutaneous rash, acute kidney injury, and temporal relationship to a high-risk drug, a diagnosis of DRESS syndrome was strongly suspected.

A skin biopsy of the rash was performed and demonstrated a superficial perivascular lymphocytic infiltrate with scattered eosinophils, consistent with a drug-induced hypersensitivity reaction.

Based on clinical, laboratory, and histopathological findings, the patient obtained a RegiSCAR score of 7, establishing the diagnosis of definite DRESS syndrome. The score reflected the presence of fever (+1), marked eosinophilia (+2), skin rash suggestive of DRESS (+1), visceral organ involvement (+1), skin biopsy compatible (+1), and exclusion of alternative etiologies (+1).

**Figure 3:**
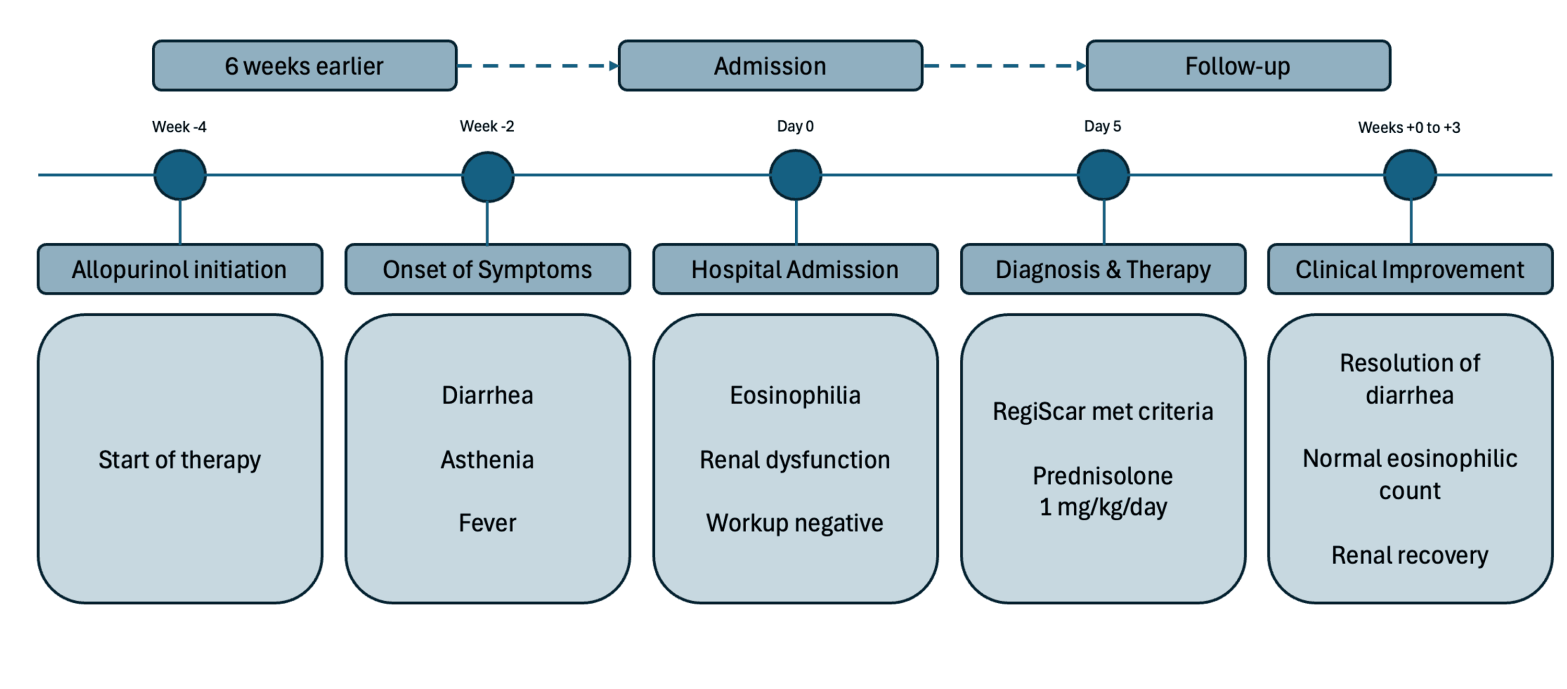
Timeline of clinical course and management The patient initiated allopurinol therapy six weeks before hospital admission. Two weeks later, symptoms including diarrhea, asthenia, and fever developed. On admission (day 0), laboratory evaluation revealed eosinophilia and renal dysfunction, while the infectious and autoimmune workup was negative. On day 5, the diagnosis of DRESS syndrome was established based on RegiSCAR criteria (7 points), and treatment with prednisolone (1 mg/kg/day) was initiated. During follow-up (weeks 0-3), progressive clinical improvement was observed, including the resolution of diarrhea, normalization of eosinophil counts, and recovery of renal function. DRESS: drug reaction with eosinophilia and systemic symptoms Image created by the authors using PowerPoint (Microsoft Corporation, Redmond, Washington, United States)

Allopurinol was immediately discontinued. High-dose systemic corticosteroid therapy was initiated with prednisolone at 1 mg/kg/day. The patient's diarrhea improved dramatically within 96 hours. Over the following three weeks, she achieved complete resolution of gastrointestinal symptoms, normalization of eosinophil count, and full recovery of renal function. At the three-month follow-up, the patient remained asymptomatic and experienced no recurrence. Figure 3 shows a timeline of clinical course and management.

## Discussion

DRESS is a severe, life-threatening hypersensitivity reaction characterized by a polymorphic rash, hematologic abnormalities (classically eosinophilia or atypical lymphocytosis), and internal organ involvement, typically presenting 2-8 weeks after drug initiation [1]. Our patient's clinical course, namely, systemic symptoms, eosinophilia, internal organ injury (colitis and acute kidney injury), and a latent period of approximately four weeks, fulfills the criteria for a definite case of DRESS syndrome according to the established RegiSCAR scoring system [1-5].

Allopurinol is one of the most common culprit drugs, accounting for up to 18% of cases in large prospective studies [1]. While its association with DRESS is well-established, often involving the liver and kidneys (interstitial nephritis) [6,7], this case highlights an unusually severe and atypical initial presentation. The patient was admitted in hypovolemic shock due to profound fluid loss from the severe colitis. DRESS syndrome carries a significant mortality rate (up to 10%), primarily related to multi-organ failure [1,5]. Our patient's initial critical presentation, marked by shock and acute kidney injury secondary to volume depletion, underscores the potential for DRESS to rapidly destabilize a patient through its systemic inflammatory and organ-damaging effects, demanding immediate critical care intervention.

The diagnostic breakthrough was the histopathology revealing severe eosinophilic colitis. Primary eosinophilic gastrointestinal disorders, inflammatory bowel disease, and parasitic infections were considered in the differential diagnosis but were excluded based on clinical presentation, laboratory evaluation, and histological findings. Gastrointestinal involvement, excluding common hepatic injury, is considered a less frequent manifestation of DRESS [3]. A recent systematic review focusing on these lesser-known gastrointestinal manifestations reported only 21 patients who developed colitis during DRESS syndrome [3]. While the colonic biopsies in these cases often showed a lymphocytic and eosinophilic infiltrate, making our patient's diagnosis histologically consistent, the clinical severity and presentation are what make this case exceptional. In patients with allopurinol-induced DRESS, the overwhelming majority of visceral cases involve nephritis or hepatitis [5,7]. Presenting with life-threatening diarrheal illness and shock, with colitis as the leading organ manifestation, represents an extraordinarily rare initial phenotype of allopurinol-induced DRESS.

## Conclusions

This case serves as a critical diagnostic alert. Following an exhaustive workup excluding infectious and common inflammatory etiologies, the finding of eosinophilic colitis in the context of recent allopurinol initiation, even with a subtle rash, must prompt immediate consideration of DRESS syndrome. Early diagnosis and prompt discontinuation of the offending drug are lifesaving, followed by high-dose systemic corticosteroids, as successfully administered in this case.
